# Cutaneous Larva Migrans Acquired in a Tropical Area of Ecuador: Diagnostic Delay, Clinical Evolution, and Recognition Challenges

**DOI:** 10.3390/tropicalmed11060155

**Published:** 2026-06-08

**Authors:** Verónica Salomé Sánchez-Peralta, Katherine Lizeth Moposa-Balarezo, Fabio Marcelo Idrovo-Espín, Rommy Terán

**Affiliations:** 1 Laboratorio de Parasitología, Facultad de Ciencias Químicas, Universidad Central del Ecuador, Francisco Viteri s/n y Gilberto Gatto Sobral, Quito 170521, Ecuador; vssanchez@uce.edu.ec (V.S.S.-P.); klmoposa@uce.edu.ec (K.L.M.-B.); 2 Laboratorio de Bioquímica LB-FCQ CL2-1, Facultad de Ciencias Químicas, Universidad Central del Ecuador, Francisco Viteri s/n y Gilberto Gatto Sobral, Quito 170521, Ecuador; fmidrovo@uce.edu.ec; 3 Laboratorio de Microbiología, Facultad de Ciencias Químicas, Universidad Central del Ecuador, Francisco Viteri s/n y Gilberto Gatto Sobral, Quito 170521, Ecuador

**Keywords:** cutaneous larva migrans, hookworm infections, neglected diseases, diagnosis, zoonoses, McNemar test, One Health, Ecuador

## Abstract

Cutaneous Larva Migrans (CLM) is a neglected tropical disease (NTD) caused by zoonotic Ancylostomatidae larvae, mainly *Ancylostoma braziliense* and *Ancylostoma caninum*, which infect dogs and cats. Humans are accidental hosts, acquiring infection when L3 larvae in contaminated soil penetrate the skin, producing serpiginous, pruritic lesions. We report a 24-year-old female from Quito, Ecuador, who developed a pruritic lesion on her right foot nine days after walking barefoot on wet, potentially fecally contaminated sand at Atacames Beach. Initial self-treatment with benzyl benzoate and herbal washes, followed by misdiagnoses as scabies and plantar warts, delayed proper care. Lesions progressed over three weeks with intense pruritus and functional impairment. CLM was correctly diagnosed by a podiatric technician 26 days post-exposure. Oral albendazole (400 mg/day for 4 days) led to rapid symptomatic relief within three days, with complete resolution by day 50. A survey analyzed by the McNemar Test revealed difficulties in recognizing early-stage CLM, regardless of experience or region among participants. Prevention requires personal protection, environmental sanitation, and regular anthelmintic treatment of dogs and cats. This case underscores the clinical consequences of delayed or incorrect diagnosis and highlights the need for enhanced healthcare training and One Health measures to reduce zoonotic diseases in Ecuador.

## 1. Introduction

Cutaneous larva migrans (CLM) is a parasitic zoonosis caused by geohelminths of the family Ancylostomatidae, mainly of the genera *Uncinaria* and *Ancylostoma*, which typically infect dogs and cats as definitive hosts. The World Health Organization (WHO) recognizes it as one of the Neglected Tropical Diseases (NTD), primarily prevalent among communities in tropical areas with limited sanitary conditions [1].

This larva develops from eggs of the parasite Ancylostoma deposited in the soil through the feces of infected dogs and cats. The rhabditiform larvae (L1) hatch into the environment, and later evolve into infective filariform larvae (L3) present in moist and contaminated soils [2]. The infective L3 larvae of Ancylostoma penetrate the skin through the stratum corneum or hair follicles, secreting zinc-dependent proteolytic enzymes such as MTP-1 that degrade connective tissue and facilitate entry. This enzymatic activity allows the larvae to migrate within the skin, causing characteristic lesions [2,3].

Although humans act as accidental hosts and the parasite does not complete its life cycle, the infestation generates bothersome clinical manifestations that affect quality of life and require correct diagnosis and timely treatment [4]. The parasites most frequently implicated in this disease are *Ancylostoma braziliense* and *Ancylostoma caninum*, with occasional cases caused by *Uncinaria stenocephala* and *Bunostomum phlebotomum*. Among these, *A. braziliense* predominates in human transmission. Notably, *Ancylostoma ceylanicum* has recently been detected in both domestic and wild dogs, representing an emerging zoonotic risk to humans across the Americas, including Ecuador [2].

Our goals were to document the case of a 24-year-old patient with CLM acquired on an Ecuadorian beach, highlighting the clinical course, risk factors, therapeutic measures, and preventive measures. Additionally, we aimed to evaluate the recognition accuracy of health professionals and students in Ecuador regarding this disease at an early stage.

## 2. Case Report

### 2.1. Clinical Features and Management

A 24-year-old female university student from Quito, capital of Ecuador, with no relevant medical history and no pets at home, presented with serpiginous skin lesions measuring 0.5 cm in width and 3.0 cm in length on the dorsum of the right foot, evolving over three weeks. The lesions were associated with intense pruritus, signs of inflammation, and functional limitation of the affected lower limb.

**Figure 1:**
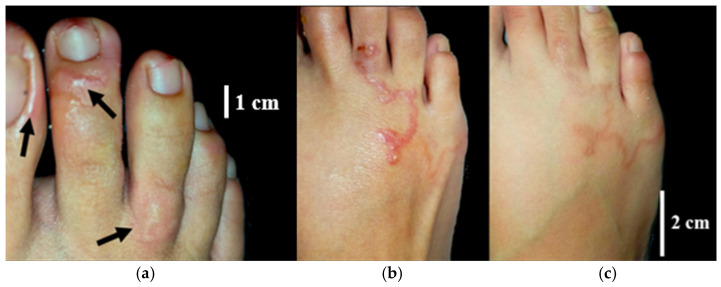
Evolution of CLM lesions: (**a**) early form of the lesion, 17 days after infection or presence of main symptoms, the black arrows show the main lesions (**b**) disease progression, 26 days after infection and treatment with salicylic acid + lactic acid (**c**) 50 days after the infection, evaluation after the treatment with albendazole. Pictures provided by the patient.

As part of the epidemiological history, the patient reported having traveled to Atacames Beach (Esmeraldas Province, Ecuador: 0°57′ N, 79°50′ W) during the last week of May 2025. On the day of exposure, after an afternoon drizzle, she walked barefoot on wet sand. The area had a foul odor, suggesting possible fecal contamination. Nine days later, she started experiencing intense itching localized on the second and third toes of her right foot. The initial lesions looked like insect bites (ants or fleas), resembling an erythemtous papule measuring 1 to 2 mm in diameter, later developing into grouped erythematous vesiculobullous lesions with a progressive distribution pattern. The lesion progressed in a serpiginous pattern around the toes, accompanied by pruritus, erythema, edema, and functional impairment (Figure 1a: 17 days after infection).

In a first home-based therapeutic attempt prompted by suspected scabies, the patient self-applied topical benzyl benzoate for three days along with hot water and chamomile (“manzanilla”, *Matricaria chamomilla*) washes, without clinical improvement. Three days later, she visited a general practitioner who misdiagnosed the lesions as plantar warts and prescribed Acyclovir 400 mg every 4 h and topical salicylic acid + lactic acid every 12 h. Over the following week, the lesions extended to approximately 20 cm, with increased pruritus and impaired mobility due to pain and local inflammation. On day 26 (Figure 1b), the patient consulted a podiatry technician who, based on the clinical presentation and history of exposure in an endemic area, diagnosed CLM and recommended Albendazole 400 mg/day for four days. After three days of treatment, there was a reduction in pruritus and stabilization of lesion progression, leading to clinical improvement. The lesion subsequently resolved into a scar, and the infection completely resolved after 50 days (Figure 1c). On day 50, a complete blood count showed leukocytes 5700/µL, neutrophils 2960/µL (52%), lymphocytes 2220/µL (38.9%), eosinophils 60/ul 1,1%, hemoglobin 13.2 g/dL, hematocrit 36.8%, mean corpuscular volume 86.6 fL, and platelets 344,000/µL, all within normal ranges. No additional symptoms were reported.

### 2.2. Assessment of Accuracy in Recognizing the Clinical Condition

Due to the difficulties in early diagnosis that contributed to the progression of the clinical condition in this case, a survey (with informed consent obtained from all participants) was conducted among individuals associated with the Ecuadorian health system to evaluate the accuracy of recognition of this “forgotten” disease. Participants (specialist and general physicians, biochemists, medicine and biochemistry students, clinical laboratory personnel, and others) were shown the early form of the lesion (Figure 1a) and the disease progression (Figure 1b). Then, they were asked to indicate the most probable diagnosis among six plausible diseases (bacterial cellulitis, contact dermatitis, scabies, CLM, tinea pedis, or plantar warts), considering the geographic region where they work in Ecuador (Highlands, Coast, Galápagos, and Ecuadorian Amazon) and their years of experience (<5, 5–10, 11–20, and >20). The responses obtained were analyzed using the McNemar test and chi-square test [5] using R [6].

Of the 72 participants, most were located in the Highlands (n = 57), followed by the Coast (n = 5), the Ecuadorian Amazon (n = 3), and Galápagos (n = 1). Regarding the participants, 27 were specialists and general physicians, 22 were students (medicine and biochemistry), 9 were biochemists, 8 were clinical laboratory personnel, and 6 belonged to other professions.

The results of the McNemar test are detailed in Table 1. The chi-square value was 41.00 with one degree of freedom and *p* = 1.522 × 10^−10^. Therefore, there is a highly significant difference in recognition accuracy among the participants when observing the initial image and the image showing disease progression.

On the other hand, no significant associations were found between geographic region (*p* = 0.2854), participant (*p* = 0.6474), or years of experience (*p* = 0.2015) and the recognition accuracy.

## 3. Discussion

### 3.1. Clinical Course

The present report illustrates a typical case of CLM after exposure to contaminated beach sand, a well-established route of transmission reported in tropical coastal environments. Infection by hookworms is among the most common soil-transmitted helminthiases, with 1.5 billion cases worldwide, causing 65,000 deaths per year. It belongs to the group of neglected diseases recognized by the WHO [1]. This condition is prevalent in places with adequate humidity and a lack of environmental sanitation. In humans, hookworms can cause systemic diseases such as malnutrition, anemia, and CLM, which represents a serious public health problem [2].

In humans, the incubation period of CLM is usually one to six days after exposure to infective (L3) larvae of *Ancylostoma*, and the clinical manifestation is an itchy, erythematous, serpiginous tract that typically appears days (or even months) after exposure. The eruption usually lasts between two and eight weeks, although cases persisting for up to two years have been reported [7,8]. The serpiginous lesions advance approximately 1–2 cm per day and reach lengths of 15–20 cm [9]. As observed, this pattern corresponds with the progression of the disease reported by the patient.

Among the most notable etiological agents of this parasitic disease is *A. braziliense*, which is associated with more intense clinical manifestations, classic migratory larvae, and characteristic serpiginous eruption. *Ancylostoma* infections in humans can range from mild to more complex forms. The response to larvae of *A. ceylanicum*, *A. caninum*, and *U. stenocephala* usually produces mild cases that resolve spontaneously within a few days. However, infections caused by the adult forms of *A. caninum* may result in symptomatic eosinophilic enteritis [10,11,12] while *A. ceylanicum* has the potential to produce more pronounced clinical symptoms, including epigastric pain, diarrhea, anemia, anorexia, malnutrition, hemorrhagic diarrhea, and other disorders such as aphthous ileitis [13,14]. In the present case, the intense serpiginous lesion symptoms could be more closely associated with *A. braziliense*, and due to the absence of digestive symptoms and anemia, it is less likely that *A. caninum* or *A. ceylanicum* were involved. Moreover, the recognition of the parasite’s early-stage characteristics by personnel within the Ecuadorian healthcare system remained limited. Despite its prevalence in our country and its classification as a neglected disease, it was often not considered in basic clinical diagnosis.

### 3.2. Risk Factors

The population at greatest risk for *Ancylostoma* infections includes children and people who walk barefoot on sandy soils (rural or beach areas) contaminated with the feces of cats and dogs that carry parasite eggs. The most common and pathogenic nematode of cats and dogs worldwide is *Ancylostoma caninum*, together with *A. ceylanicum*, *A. brasiliense*, and, to a lesser extent, *Uncinaria stenocephala*; these are the causes of cutaneous larva migrans in humans [2,9,15]. *A. braziliense* is associated with areas where large numbers of dogs and cats live in overcrowded conditions. *A. caninum* is predominant in Australia, while *Uncinaria stenocephala* is found in Europe [16].

Studies conducted in coastal areas and beaches of Ecuador reported a prevalence of 62% for these parasitic diseases in cats and dogs; of these infections, 47.5% were attributed to *Ancylostoma* spp., followed by *Toxocara* and *Dipylidium caninum* [17]. Furthermore, in the same region, an *Ancylostoma* prevalence of 50.20% in canines was reported, similar to that observed in urban and rural areas worldwide, although lower than the 60% prevalence found in endemic regions of South America [18].

In addition, according to the National Institute of Meteorology and Hydrology (INHAMI), the meteorological station MO0058 at the Coronel Carlos Concha Torres International Airport, reported by the end of May 2025, an accumulated precipitation of 32.30 mm in the province of Esmeraldas, with surface humidity classified as dry and temperatures within the normal range (around 25 °C) [19]. Considering the drizzle that occurred on the day of exposure and the possible fecal contamination of the area, it is plausible that these factors created an optimal environment for the presence and survival of *Ancylostoma* spp. larvae originating from the tropical fauna at Atacames Beach, Ecuador, facilitating contact with the patient’s unprotected skin.

### 3.3. Therapeutic Measures

In clinical practice, the most commonly used oral regimens are albendazole and ivermectin, which help reduce symptoms and prevent bacterial superinfection. Ivermectin is administered at 200 mcg/kg orally once daily for one to two days. It is well tolerated and has cure rates ranging from 94 to 100%. Albendazole may also be used at a dose of 400 mg every 12 h for three days [10]. With both medications, symptoms usually resolve within a few days after starting antiparasitic treatment and rarely persist beyond one week. In the present case, the patient received albendazole 400 mg daily for four days, with progressive clinical improvement, consistent with the rapid response expected according to the literature, and did not require additional treatments such as antihistamines or corticosteroids. Symptoms usually resolve within a few days after starting antiparasitic treatment and rarely persist beyond one week.

Topical formulations are also available, including 10% albendazole ointment, 10–15% thiabendazole dermal cream, and 10–15% aqueous suspensions, demonstrating up to 98% efficacy [20]. However, these formulations are not available in Ecuador.

The patient reported using chamomile as an initial self-treatment. However, this approach is not appropriate, as there is no evidence to support its efficacy in eliminating CLM. Nevertheless, the use of traditional herbal medicine is well documented in rural communities and coastal regions in Ecuador, where it is commonly employed to manage digestive, infectious, and parasitic conditions [21].

### 3.4. Preventive Measures

Anthelmintic treatment is crucial in dogs and cats, the main reservoirs of hookworm larvae. Puppies are particularly susceptible to transplacental or transmammary infection and require treatment at two, four, six, and eight weeks. Pregnant dogs should receive treatment from day 40 of gestation to day 14 of lactation to reduce transmission to offspring. Regular veterinary checks, including fecal flotation, Polymerase Chain Reaction (PCR), or parasite-specific antigen tests, are essential to detect infections [22].

Puppies should be tested at least four times in the first year, while adult dogs benefit from semiannual testing depending on health and lifestyle. *A. caninum* and *A. tubaeforme* larvae feed on blood before the adults produce eggs, so puppies infected via maternal lactation may develop acute anemia or die before eggs appear in feces [22]. Preventive measures in animals include controlling scavenging behaviors and removing dog and cat feces daily. Regular anthelmintic treatments, veterinary care, and owner education are critical, especially in resource-limited areas with stray animals [22,23]. In humans, prevention focuses on avoiding contact with contaminated soil or sand by wearing shoes, gloves, or protective clothing, and using towels or chairs on beaches. Covering sandboxes and playgrounds and properly disposing of feces reduces exposure [23]. Combined veterinary care, anthelmintic treatment, environmental hygiene, and personal protection prevent hookworm-related CLM [22,23]. This integrated strategy reflects the One Health approach, recognizing the interconnected health of humans, animals, and the environment in controlling zoonotic diseases such as CLM. On the Ecuadorian coast, preventing CLM should require wearing appropriate footwear, maintaining clean beaches and playgrounds, and ensuring regular anthelmintic treatment of dogs and cats. Combined with personal hygiene and veterinary oversight, these measures would significantly reduce larval transmission.

### 3.5. Recognition Accuracy Survey

The McNemar test results show that the early form of the lesion (Figure 1a) is difficult to recognize clinically. This was evident in the patient, who initially self-medicated and subsequently received an incorrect diagnosis from a physician before receiving appropriate treatment. In Ecuador, diagnostic challenges are common for tropical diseases, especially where exposure to these diseases is limited. Previously, a case of babesiosis (the second case reported in Ecuador) highlighted underdiagnosis due to limited knowledge among healthcare personnel, lack of epidemiological studies, and insufficient availability of sensitive diagnostic methods; even PCR or serology can fail to detect the disease, with sequencing being the most reliable method [24]. Additionally, lymphatic filariasis in Ecuador was considered underdiagnosed, with errors in case registration and clinical diagnosis, especially at the primary care level and during medical student training in Quito (the capital of Ecuador, located in the Highlands) [25]. These findings underline the overall challenges in achieving recognition accuracy for tropical pathologies, even for conditions like CLM, which should be relatively easier to identify and manage in our country, where studies have documented the endemic presence of *Ancylostoma* spp. in urban animal populations along the coastal regions [2,15,16].

### 3.6. Conclusions

This case illustrated a typical presentation of CLM following exposure to contaminated beach sand in a tropical coastal environment. The serpiginous lesions, intense pruritus, and lesion progression were most consistent with *A. braziliense* infection, while the absence of gastrointestinal symptoms or anemia made involvement of *A. caninum* or *A. ceylanicum* unlikely.

Early recognition of CLM remained limited among healthcare personnel in Ecuador, contributing to misdiagnosis and delayed treatment. The recognition accuracy survey confirmed that early lesions are challenging to identify, regardless of participants’ geographic location, professional background, or experience. Prompt administration of albendazole led to rapid clinical improvement, highlighting the effectiveness of standard anthelmintic therapy. Traditional home remedies were ineffective.

Preventive measures, including wearing protective footwear, maintaining environmental sanitation, and regular anthelmintic treatment of domestic animals, are essential to reduce transmission in endemic areas.

The integration of the One Health approach is essential for understanding CLM, as it considers the interconnected relationship between human, animal, and environmental health. The presence of infected dogs and cats, inadequate management of fecal waste, and human exposure to contaminated soil create a transmission cycle that can be prevented through coordinated control measures and public health education. This study emphasizes the need for surveillance and prevention strategies at beaches and recreational areas of endemic regions, as well as the education of tourists and local communities about exposure risks. This report underscores the need for increased awareness and training among healthcare providers to ensure early diagnosis and effective management of CLM, a neglected but prevalent tropical disease in Ecuador.

### 3.7. Study Limitations

The lack of significance between geographic region and recognition accuracy may be related to the fact that most participants were from the Highlands, where they would have had limited familiarity with this pathology during both medical training and professional practice. Therefore, for future studies, it is necessary to increase the number of participants working in the Coast and other warm-climate areas where clinical experience with this disease is more likely.

## Figures and Tables

**Table 1:** McNemar test contingency table comparing the recognition accuracy between the early form of the lesion (Figure 1a) and the disease progression (Figure 1b).

	Succeeded	Failed
Succeeded	20	0
Failed	41	11

## Data Availability

The original contributions presented in this study are included in the article. Further inquiries can be directed to the corresponding author.
